# Conceptualizing an Interdisciplinary Collective Impact Approach to Examine and Intervene in the Chronic Cycle of Homelessness

**DOI:** 10.3390/ijerph18042020

**Published:** 2021-02-19

**Authors:** Mounah Abdel-Samad, Jerel P. Calzo, Jennifer K. Felner, Lianne Urada, Matthew E. Verbyla, Hala Madanat, Brian E. Adams, Thais Alves, Bruce Appleyard, Joshua Chanin, Shawn Flanigan, Hisham Foad, Maya Ginsberg, Matthew Higgins, Eunjeong Ko, Kristen Maher, Natalie Mladenov, Peggy Peattie, Megan Welsh, David Sleet

**Affiliations:** 1 School of Public Affairs, San Diego State University (SDSU), San Diego, CA 92182, USA; bappleyard@sdsu.edu (B.A.); jchanin@sdsu.edu (J.C.); shawn.flanigan@sdsu.edu (S.F.); mwelsh@sdsu.edu (M.W.); 2 School of Public Health, SDSU, San Diego, CA 92182, USA; jcalzo@sdsu.edu (J.P.C.); jfelner@sdsu.edu (J.K.F.); hmadanat@sdsu.edu (H.M.); 3 School of Social Work, SDSU, San Diego, CA 92182, USA; lurada@sdsu.edu (L.U.); eko@sdsu.edu (E.K.); 4 Department of Civil, Construction, and Environmental Engineering, SDSU, San Diego, CA 92182, USA; mverbyla@sdsu.edu (M.E.V.); talves@sdsu.edu (T.A.); nmladenov@sdsu.edu (N.M.); 5 Department of Political Science, SDSU, San Diego, CA 92182, USA; badams@sdsu.edu (B.E.A.); kmaher@sdsu.edu (K.M.); 6 Department of Economics, SDSU, San Diego, CA 92182, USA; hfoad@sdsu.edu; 7 School of Music and Dance, SDSU, San Diego, CA 92182, USA; mginsberg@sdsu.edu; 8 School of Art and Design, SDSU, San Diego, CA 92182, USA; mhiggins@sdsu.edu; 9 School of Journalism and Media Studies, SDSU, San Diego, CA 92182, USA; ppeattie@sdsu.edu

**Keywords:** homeless, homelessness, interdisciplinary, academic-practice, collective impact model

## Abstract

Homelessness is a persistent problem in the United States in general and in Southern California especially. While progress has been made in reducing the number of people experiencing homelessness in the United States from 2007 (647,000) to 2019 (567,000), it remains an entrenched problem. The purpose of this paper is to outline a novel, interdisciplinary academic-practice partnership model to address homelessness. Where singular disciplinary approaches may fall short in substantially reducing homelessness at the community and population level, our model draws from a collective impact model which coordinates discipline-specific approaches through mutually reinforcing activities and shared metrics of progress and impact to foster synergy and sustainability of efforts. This paper describes the necessary capacity-building at the institution and community level for the model, the complementary strengths and contributions of each stakeholder discipline in the proposed model, and future goals for implementation to address homelessness in the Southern California region.

## 1. Introduction

Homelessness is a persistent problem in the United States (US). The Housing and Urban Development (HUD) point in time count, which is one approach for tracking the prevalence of homelessness in the US, indicates an overall 15% decline in the number of persons experiencing homelessness from 2007 (approximately 647,000) to 2019 (approximately 567,000) [1]. This decline can be attributed to the strong focus on homeless reductions in subgroups—such as veterans and families—who saw substantial declines since 2007 (38% and 23%, respectively). Nevertheless, the number of individuals experiencing homelessness in the US remains stubbornly high. It is anticipated that with COVID-19, gains made in homelessness reduction will be lost and disparities among some of the most vulnerable groups, such as racial and ethnic minorities, will widen [2,3,4,5].

While research efforts continue to evaluate and identify practices and interventions that mitigate the harms of experiencing homelessness and reduce the risk of chronic homelessness (e.g., Housing First approaches and Rapid Rehousing interventions [6,7,8,9]), the persistently high prevalence of homelessness across the US indicates the need for continued work to develop, study, and implement successful strategies to prevent homelessness. Sustainable solutions to this “grand challenge” [10] cannot be developed by teams working within disciplinary silos; they require an interdisciplinary systems perspective that appreciates the complexity of the challenge [11,12].

The purpose of this paper is to outline a novel, interdisciplinary academic-practice partnership model to address homelessness. Where singular disciplinary approaches may fall short in substantially reducing homelessness at the community and population level, our model draws from a collective impact model which coordinates discipline-specific approaches through mutually reinforcing activities and shared metrics of progress and impact to foster synergy and sustainability of efforts. This paper describes the necessary capacity building at the institutional and community level for the model, the complementary strengths and contributions of each stakeholder discipline in the proposed model, and goals for future implementation to address homelessness in the Southern California region. California has the highest population of homelessness in the country and the San Diego region located in Southern California has the fourth largest population of people experiencing homelessness among U.S. cities and regions [13].

### Overcoming Decentralized Approaches to Homelessness through a Collective Impact Model

People at risk for or experiencing homelessness must navigate multiple systems (e.g., public health, medicine, social work) to fulfill their most basic needs, and live in and traverse social and built environments that are shaped by cultural norms (e.g., altruism, stigmatization of homelessness) and public policies (e.g., laws against loitering, zoning for shelters) that constrain their social and economic mobility [14,15,16]. Despite the complexities and interrelatedness of the determinants that create and perpetuate homelessness, traditional strategies to address homelessness typically focus on one aspect of the lived experiences of homelessness (e.g., availability of shelter beds and affordable housing). Very few programs or policies simultaneously address preventative measures (i.e., explicitly focused on upstream causes of homelessness) while also reducing the chronic cycling of homelessness. In addition, people experiencing homelessness are not often included or consulted in devising efforts to develop programs and policies that impact their lives. Furthermore, academics, policymakers, and other agents of change often operate in silos rather than across disciplines.

We argue, here, for a unified, interdisciplinary approach to preventing and mitigating the impacts of homelessness using a collective impact model. According to Kania and Kramer, “collective impact initiatives involve a centralized infrastructure, a dedicated staff, and a structured process that leads to a common agenda, shared measurement, continuous communication, and mutually reinforcing activities among all participants” ([17], p. 38).

**Figure 1:**
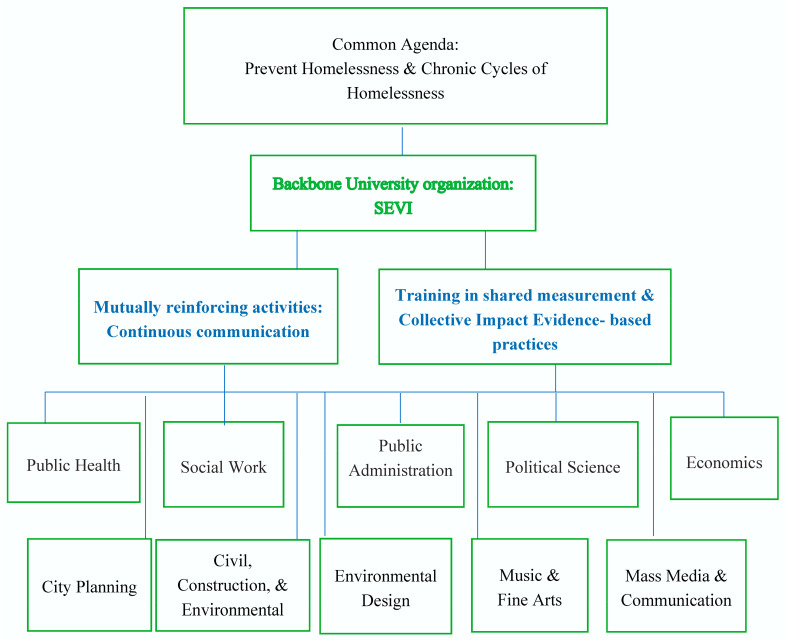
Three components of an interdisciplinary collective impact academic-practice partnership.

Drawing from the collective impact model, we highlight a strategy that unifies disciplinary approaches and efforts under a common organizational framework, in our case, the Social and Economic Vulnerabilities Initiatives (SEVI) at San Diego State University (SDSU, see Figure 1). This approach centralizes information, supports collaboration, and monitors progress on addressing different phases of homelessness through shared metrics and mutually reinforcing activities [17,18]. At SEVI, we have created the groundwork for such a coordinated approach by developing an academic-practice partnership. In addition to coordinating multi-disciplinary research teams within the university, SEVI collaborates with other universities, service providers, and with local government agencies to target research toward specific topics of interest to local stakeholders. The objective of this collaboration is to create through meetings, surveys, and one-on-one meetings a shared agenda based on input from research teams, service providers, and local government agencies.

This partnership incorporates the disciplines of public health, social work, public administration, political science, engineering, city planning, economics, mass media and communication, fine arts, and design (see description of unique disciplinary stakeholder engagement around homelessness in Section 2.2). Faculty and students from these disciplines work in partnership with policymakers, public institutions (e.g., county public health, local cities, county educational and housing departments), individuals who have experienced or currently are experiencing homelessness, and service providers (e.g., federally qualified health centers, and community non-profit institutions such as the YMCA, Catholic Charities, People Assisting the Homeless [PATH], Think Dignity), creating a collaborative co-learning environment. This environment expedites the translation of research into action and facilitates the evaluation, innovation, and dissemination of effective community practices.

The implementation of this model has financial, institutional, and human resource challenges. Universities implementing this model need to invest financially in their backbone organization to build capacity to coordinate community and researcher collaborations. In addition, universities need to invest in building institutional processes that simplify collaboration, agenda setting, and evaluation. Finally, since each faculty member and community entity are independent and following their own agenda, directors of such initiatives need to build personal and professional relationships that increase the success of such collaborations.

However, we contend that such a model, supported by proper university infrastructure, can improve understanding of how different disciplines can integrate efforts to address homelessness in all its phases (pre-homelessness, homelessness, and post-homelessness). The coordination of community-academic efforts across disciplines through a single hub using shared metrics can help to contribute to several interrelated efforts. These efforts include direct implementation and refinement of short and long-term preventative measures to reduce the incidence and prevalence of homelessness, reduction of risk of chronic cycling of homelessness, mitigation of the long-term public health impact of homelessness, and identification of emergent programmatic priorities by working directly with people experiencing homelessness and community stakeholders.

There are three components to our collective impact model for an interdisciplinary academic-practice partnership.

1. Make evidence-based decisions based on a common agenda, integrating knowledge across disciplines: Our first component takes the form of building a more comprehensive approach to addressing homelessness through leveraging evidence-based approaches from multiple disciplines and translating approaches across disciplines to prevent homelessness and the chronic cycling of homelessness.
2. Use community-based participatory approaches through mutually reinforcing activities and continuous communication feedback loops: Our approach invests in the use of community-based participatory approaches, which undergird the academic-practice partnerships. By partnering directly with people experiencing homelessness, as well as with policymakers, public institutions, and community practitioners, we not only enhance understanding of how to prevent and reduce homelessness on a local level, but also build the capacity of communities to sustain prevention efforts.
3. Train a 21st century workforce who are skilled in interdisciplinary approaches: The partnership will help develop the workforce of tomorrow (our SDSU students), who will develop the skills to understand, mitigate, and prevent homelessness. Faculty engage students in funded research and develop students’ analytical skills and knowledge.

## 2. An Interdisciplinary Academic-Practice Partnership Model

### 2.1. Preventing Homelessness and Disrupting the Chronic Cycle of Homelessness Via an Interdisciplinary Academic-Practice Partnership Model

The experience of homelessness can be conceptualized as a continuum, from pre-homelessness, to homelessness, to post-homelessness, with some individuals experiencing recurring cycles of homelessness and post-homelessness (i.e., the cycle of homelessness). Our model can be applied across the continuum of homelessness, with the goal of leveraging the complementary strengths of multiple disciplines and the overall academic-practice partnership to prevent individuals from experiencing homelessness entirely, and/or disrupting the chronic cycle of homelessness. Our model builds a common agenda across disciplines that focuses on homelessness in general terms.

Throughout different stages of homelessness, our model encourages faculty and collaborators to reinforce each other’s activities by supplementing data and engaging in new research questions. For example, researchers, service providers, people experiencing homelessness, public institutions, and policy makers, reinforce each other’s activities by examining shared questions. As part of SEVI’s mission, we allocate resources to support research projects grounded in a common research agenda.

In addition, by developing and incorporating the shared-metrics-principle of the collective impact approach, collaborators from different disciplines have a common set of metrics. For example, a common metric of success may not be simply how many individuals/families have been housed, but also how many housed individuals/families are economically secure and are physically and mentally healthy.

#### 2.1.1. Pre-Homelessness Phase 

There are several concrete ways that different disciplines can coordinate efforts to address homelessness at the pre-homelessness phase. For example, new preventative policies may be introduced that affect public health, housing, and city planning. In this phase, various disciplines can examine public opinion about ways to prevent homelessness (e.g., not-in-my-backyard [NIMBY]ism), detect obstacles to policy making, accelerate effective budgetary decision-making, identify ways to address factors in the criminal justice and foster care systems that increase homelessness, examine alternatives to transportation for individuals experiencing homelessness, and address housing shortfalls.

#### 2.1.2. Homelessness Phase 

In this phase, collaborations between economics, public administration, political science, and public health, along with service providers and public institutions, allows our academic-practice partnership to examine and evaluate the success of various interventions (e.g., in mental health). In this case, the disciplines explore the same questions from different perspectives to understand how to improve services or prevention. For example, the school of music, social work, public health, and public administration could collaborate to explore the role of music and art programs in improving mental health. Music programs can build a sense of community among their members, reduce anxiety, and enhance mental health of participants. In addition, criminal justice, social work, engineering, and public health could collaborate to examine the criminalization of homelessness and the opportunity to use social workers as an adjunct to police services, as well as alternatives to police intervention.

#### 2.1.3. Post-Homelessness Phase 

This phase focuses on people who have experienced homelessness after they are housed. Once housed, these individuals or families often face the same factors that may have led them to experience homelessness in the first place—for example, food insecurity, discrimination, inadequate healthcare coverage, or substance abuse. Being able to navigate these challenges and stay housed is essential in order to reduce risk for the chronic cycling of homelessness. Understanding what factors may reduce risk for chronic cycling of homelessness can be critical to the success of housing individuals after being homeless.

Similar to the previous phases, many disciplines can play an important role in understanding and proposing solutions to prevent chronic cycling of homelessness. Environmental design and engineering can play an essential role in creating living spaces that ensure affordable access to housing, improving mental health by having open green spaces, and ensuring access to health and social services. In addition, music, business, and social work can ensure there are interventions that stabilize individuals’ lives and reduce chronic cycling of homelessness.

Public administration, economics, public health, and criminal justice can collaborate to identify strategies to reduce the chances of formal and informal eviction, hence trying to improve the chances of keeping people housed. For example, by focusing on economic and mental and physical health factors, and on the interaction between citizens and police, different disciplines might provide mitigating strategies to improve the chances of people staying housed.

#### 2.1.4. Community Voices 

Throughout these phases, our collaboration focuses on engaging individuals facing homelessness, public institutions, service providers, and policy makers. Following community-based participatory approaches [19,20], our collaboration examines areas of needs, explores gaps in research, and aims to develop a program of research and practice that is responsive to local communities. Indeed, evidence suggests that engaging local communities and businesses in the development of prevention and intervention strategies, and in the implementation of those strategies, leads to greater long-term success than not involving community and considering local culture [20,21,22].

### 2.2. Preliminary Applications of the Interdisciplinary Academic-Practice Partnership to Address Homelessness in San Diego

Currently, efforts by SDSU faculty and students to study and intervene in homelessness in San Diego County do not fully utilize all aspects of the collective impact model, nor do they realize the maximum potential of interdisciplinary collaboration. However, as described by the following vignettes, there is great potential for synergy and expansion of work. We invite readers to consider how they might collaborate with other allied disciplines and community partners to enhance the impact of their work on homelessness.

#### 2.2.1. Vignette 1

In 2018–2019, two of the authors (J.K.F. and J.P.C.) conducted a community-based participatory research study using photovoice (i.e., photography, videography, and critical narratives-as-data) and complementary qualitative methods, to explore how the social and built environment shaped health among transitional aged youth experiencing homelessness in San Diego. The study and its findings are detailed elsewhere [14]. Briefly, the community-academic study team consisted of academic faculty and student research assistants from SDSU’s School of Public Health, as well as community members with lived experience with homelessness. The study team collected and analyzed data, and member-checked emergent findings with local groups of transitional aged youth experiencing homelessness and adult stakeholders. Findings were presented in a community forum with local policymakers, youth service providers, and youth experiencing homelessness, followed by an open dialogue about data-supported recommendations germane to supporting the well-being of transitional aged youth experiencing homelessness.

The study team published a community report and open-access scientific article to share the findings and recommendations broadly and made a concerted effort to disseminate the products to interested stakeholders. However, the forum and the disseminated products led to few follow-up discussions about how the study team’s recommendations could create local change. An interdisciplinary approach to the study, however, may have strengthened its potential for impact. For example, if scholars from public administration and political science (among others) had been involved as co-investigators, the study design may have included the collection of data informed by local policymakers and easily translated findings for policy change; the development of policy recommendations consistent with the local policy climate (i.e., “winnable” solutions); and the dissemination of the recommendations to policymakers with the ability and desire to enact them.

Another field that would have strengthened the study’s impact includes fine arts, journalism, and mass communication. Had scholars from these disciplines been involved as co-investigators, the photographic data collected may have had greater visual impact and been more effectively communicated to a diverse audience. Similarly, had scholars from city planning and civil, construction, and environmental engineering been involved as co-investigators, the study may have produced findings and recommendations with specific implications for altering the built environment to better support the needs of transitional aged youth experiencing homelessness. Furthermore, students from across these disciplines (and others) could have been involved as interns or research assistants, building their capacity to meaningfully contribute to future community-based efforts to address homelessness in San Diego.

#### 2.2.2. Vignette 2

In 2018–2019, two of the authors (M.A.-S. & M.W.) examined the interaction of people experiencing homelessness with police on the streets of San Diego. Based on interviews, surveys, and focus groups, the authors found that individuals experiencing homelessness perceived that police interaction with them is based on preconceived views of criminality and deviance. On the other hand, the article found that the widely used model of Homeless Outreach Teams (HOT), which are composed of mainly police officers and which aims at assisting people experiencing homelessness, lacked a clear pipeline from outreach to housing. These problems reduced individuals’ trust and willingness to accept services from the HOT team. This research was presented to the City of San Diego and published in a peer-reviewed journal [23]. While its impact is still being discussed, the combination of faculty from Criminal Justice with that of faculty from Public Administration not only allowed the researchers to examine perception but to also understand the police as an institution and how such institutions with its members have to manage expectations and roles of its members.

If our interdisciplinary model was implemented in full force, this research could have included disciplines like public health, social work, and environmental engineering. These disciplines could have shed light on the health (such as access to sustainable health services) and environmental (such as access to sanitation) factors increasing vulnerability of people experiencing homelessness and connected how satisfying these needs might have opened an opportunity for a different kind of police–homelessness interaction to take place. It might have even provided a justification for a totally different approach where social workers replace police outreach.

In addition, if economics, music, and journalism were involved in the study, the research might have found economic factors that make people more vulnerable to police interaction. Then, solutions might have been presented with musical programs that might build connections across identities and and alleviate the stress of living on the streets. Finally, journalism researchers might have been able to provide stories and understanding that present the lives of people experiencing homelessness to the police and the public, thus reducing the police preconceived perception that people experiencing homelessness are criminal and deviant.

As described in the two vignettes above, our approach has the potential to enrich our approaches to homelessness. By adding different disciplines to research projects, researchers can potentially improve their examination of the issues from different perspectives and improve policy recommendations. For example, some disciplines might focus on current individual factors affecting the lives of people experiencing homelessness; however, what might be needed is a more multidimensional longitudinal examination of factors that can give a more accurate picture about individuals and families and their vulnerabilities. By bringing together multiple disciplines, our approach combines their theories, research methods, perceptions, and frames of reference to examine and understand comprehensively the issue of homelessness.

### 2.3. Discipline-Specific Contributions to Addressing Homelessness

Each discipline brings with them new lenses by which to examine homelessness. The different disciplines below share a common purpose, but bring unique approaches to studying and addressing homelessness.

In terms of interventions, disciplines such as criminal justice, public health, social work, fine arts, and mass media have focused on examining environmental and social factors such as decriminalization of homelessness, changing the narrative concerning people experiencing homelessness, and creating programs that build resilience among people experiencing homelessness. In addition, many disciplines have also focused on examining factors affecting or causing homelessness. Each discipline examines housing from different lenses, such as its design and its connection to housed individuals, its costs, its location vis-a-vis where people work, and the impact of lack of affordable housing. Disciplines such as public administration, political science, economics, and fine arts have focused on how policies affecting individuals/families experiencing homelessness were developed and implemented and how such policies are evaluated. Finally, many disciplines such as public health, economics, engineering, and environmental design highlighted the need to engage communities in the research or intervention process to enhance results.

In the following section, we describe how different disciplines represented by the co-authors’ academic/disciplinary affiliations contribute to understanding and addressing homelessness. By highlighting the unique perspectives and contributions of each discipline, our goal is to highlight the many opportunities and benefits of interdisciplinary collaboration.

**A.** **Public Health**

With epidemiology and the promotion of health equity at its core, public health quantifies patterns and distributions of disease across populations and focuses on an ecological approach to health and well-being. [24,25]. Unaffordable housing and homelessness have been recognized as substantive public health problems by leading public health professional organizations (e.g., American Public Health Association) and federal and state governing bodies focused on securing the health of the nation (e.g., U.S. Department of Health and Human Services). This recognition relates to the dual function of housing instability and homelessness as determinants of physical and mental health problems, and outcomes resulting from myriad health problems [26,27]. In addition, research consistently finds that youth and adults from socially and economically marginalized communities experience disparities in housing stability and homelessness (e.g., [28,29]), further exacerbating the negative health and social outcomes produced by histories of structural and social inequity. Most recently, homelessness has come into sharp focus as a public health problem in relation to two infectious disease outbreaks—hepatitis A [14,30] and COVID-19 [31,32].

Fundamentally, public health researchers and practitioners are well-trained for interdisciplinary, collective impact approaches to addressing homelessness. Public health emphasizes the critical nature of engaging communities in research and intervention development for sustainable community health improvements [19]. As a result, public health funding bodies (e.g., the National Institutes of Health) are beginning to prioritize the inclusion of community partners in public health inquiry and intervention efforts. In addition, public health emphasizes coordination among policymakers and agencies at the federal, state, and local level to increase funding for evidence-based programs and practices to decrease homelessness (e.g., Housing First models) and enact structural-level changes to decriminalize homelessness, treat homelessness itself as a health problem, and develop prevention or early intervention programs to prevent chronic cycles of homelessness, particularly among priority populations such as veterans and youth [33,34,35].

**B.** **Social Work**

Social work reaches the most marginalized, vulnerable populations and collaborates with other disciplines to identify those at highest risk for or on the brink of homelessness [36]. Social workers are mental health practitioners and strengths-based case managers who provide one-on-one services to their clients, families of clients, and in groups. They are on the frontlines serving marginalized groups in child welfare, gerontology, mental health, community-based non-profits, private or county social services, and healthcare systems. As an integral part of an interdisciplinary team, social workers are also community organizers and administrators of social service entities, partnering within and across sectors around a common agenda and shared measurement definitions of homelessness. For example, they engage in mutually reinforcing activities with other sectors (e.g., law enforcement, justice systems, education, child welfare) to prevent and intervene with individuals in dire situations that may stem from or lead to homelessness (e.g., running away from domestic violence or child abuse, relapsing on substances).

Social workers, alongside community partners with lived experiences of homelessness, respond to the needs of people experiencing homelessness via outreach and crisis intervention in spaces where others may not go (e.g., street outreach, public libraries) [37,38,39], or in alternative approaches to ones that have not been successful for preventing or effectively mitigating homelessness or its associated harms. For example, police-led Homeless Outreach Teams have become a common feature in city police departments across the US. These teams typically include specially trained police officers working alongside social service providers and psychiatric clinicians.

Utilizing clinical and consensus organizing skills, social workers build trust through continuous communication with police officers and other agencies [18], usually with a backbone organization such as the SDSU Consensus Organizing Center housed in the School of Social Work. However, prior research suggests that police officers’ presence on these teams can hinder homeless outreach efforts, particularly for people experiencing chronic homelessness and/or those who may be “service avoidant” for an array of reasons, most notably past negative encounters with the police that have resulted in tickets, arrests, and the loss of personal belongings [23,40]. Social workers can be the leaders in a paradigm shift away from police as first responders to homelessness and toward a more social service-oriented and trauma-informed approach to outreach. This will not only increase the likelihood of connecting people to the services they need and increasing placements into permanent housing, but it will also reduce unnecessary police contact and the criminalization of homelessness.

**C.** **Public Administration**

The work of public administration scholars can attend to the complex and fraught experiences of individuals as they interact with public and non-profit institutions (e.g., [41,42,43,44]). Public administration scholars examine how institutions, organizations, stakeholder networks, and individual lawmakers shape efforts to prevent and mitigate homelessness. The field of public administration has contributed to understanding the effects of policies targeted toward people experiencing homelessness, including efforts to promote compliance with organizational and jurisdictional rules [45], description and analysis of the challenges faced by front line workers [46,47,48], and the factors shaping the use of discretionary authority [49,50,51].

Public administration scholars have also studied the organizational patterns of homeless service delivery. This work has shown that effective administrative outcomes are more likely to be generated by public actors working across inter-agency [52] and inter-governmental boundaries [53,54], as well as with private [55,56,57], and non-profit partners [58], highlighting the critical nature of collaborative work to address homelessness. For example, an analysis of 145 county-level strategic plans in the United States found that governments that worked in partnership with a diversity of leaders from other organizations were more effectively able to increase the number of available beds for members of their communities than governments that either did not develop strategic plans or did so without explicit collaboration [59].

Public administration is, at its core, a multidisciplinary field. Public administration scholars are trained to work with concepts drawn from other disciplines and possess knowledge that can be integrated into research from other fields and applied to problems more traditionally the domain of others. Working with scholars from public health, criminal justice, social work, and city planning, to name a few, will be more efficient and more productive with a common agenda and a shared set of goals.

**D.** **Political Science**

Political scientists have unique insights into the institutional complexity of homeless services, which an array of local, state, and federal governments and agencies provide [60]. While many government entities and agencies provide services, the fragmentation of service provision leaves space for different jurisdictions to evade responsibilities and costs, or work at cross-purposes [61,62]. Political scientists have the tools to lend insight into the bases of conflict over homelessness and homeless intervention, as well as potential paths to resolve them. For instance, the reasons residents give for their opposition to homeless-serving facilities are well documented in reports and media: they fear facilities will increase crime, act as a ‘magnet’ for more people experiencing homelessness to move to the neighborhood, lower property values, and reduce quality of life (e.g., [63,64]).

Political scientists conduct research to understand the underlying interests and rationale behind various policy positions [65,66]. In addition to exploring public attitudes towards people experiencing homelessness, political scientists examine how those experiencing homelessness value various approaches to meeting their own needs, and how those perceptions can shape the potential success of various homeless intervention strategies [64,67]. Although political science research has specialized strengths to contribute to understanding and addressing homelessness, it can benefit from an interdisciplinary academic-practice partnership model. For instance, other disciplines focus more on theories and determinants of homelessness, or intervention development and implementation. Sharing information and a common agenda would broaden the scope of political science research and improve analysis of how best to organize governmental institutions. In addition, collaboration with social workers and practitioners on the ground would lend a broader span of perspectives on how governmental structures operate, leading to more robust and nuanced policy analyses.

**E.** **Economics**

Economists have the potential to make significant contributions to the study of homelessness, both in terms of theory and empirical methodology. From a collective impact approach, involvement of economists in developing research and intervention approaches is particularly powerful in understanding issues of cost-effectiveness of solutions, distribution of economic burden, and identification and measurement of indicators of social and economic mobility.

Economists often employ longitudinal datasets that explore a cross-section of individuals measured over time. The use of these datasets that allow for both time varying determinants and individual level fixed effects are critical to assessing things such as differential effects of short vs. long-term homelessness or the relationship between substance abuse and homelessness. In addition, economists can measure the determinants of homelessness. For example, economists might study how homelessness is driven by long-run structural economic changes (e.g., automation, globalization) or if it is the result of idiosyncratic shocks (e.g., unexpected healthcare bills, loss of a spouse/partner, drug addiction, etc.).

Economic analysis is also useful for evaluating the effectiveness of different policies targeting homelessness. Kertesz and Johnson (2017) found that in 11 of 12 randomized control trials, unconditional housing vouchers led to greater housing retention than standard programs [68]. Furthermore, housing subsidies performed about the same in terms of participant’s health compared to standard programs that prioritize health. Program evaluation is another area in which economists can contribute to homelessness research. What is the cost of administering a specific program and what are the benefits? For example, Corinth (2017) examined increases in HUD funding and founds that a 100 unit increase in permanent supportive housing beds only reduced point-in-time homelessness by 10 [69]. One explanation is that beds may be targeted toward people experiencing homelessness with the most severe psychiatric or substance abuse problems. As such, the quality of supportive housing falls and the quality of shelters/encampments rises when these people are shifted from one to the other.

**F.** **City Planning**

In synergy with environmental design and engineering, city planners consider macro-level design aspects that directly and indirectly impact the continuum of homelessness. City planners deal directly with the spatial arrangements of land use activities, which includes the supply and positioning of housing and employment opportunities and transportation services. Planners deal with mechanisms to provide affordable housing and can examine the impact of transportation on people who might become vulnerable to homelessness (e.g., the location of housing relative to stabilizing and gainful employment opportunities) [70,71,72,73].

Transportation can be a major issue during both the pre- and post-homelessness phase in terms of maintaining employment [74]. During the homelessness phase, transportation is a major issue as people experiencing homelessness often need to bring their belongings with them and travel with their belongings to acquire services. Welfare-to-work research indicates that diverse transportation strategies are needed to support economic mobility (i.e., sustainability of employment and, therefore, housing) among people experiencing homelessness [75]. Planners examine the types of transport services needed, such as driving, bicycling, and walking; fixed route and fixed scheduled transit (e.g., bus, light rail); and more flexible and on-demand services like dial-a-ride and ride-hailing (e.g., Uber and Lyft). City planners must collaborate with other disciplines to enhance understanding of how people access key services, and maintain control of their belongings as they traverse space and the continuum of homelessness. Critical for collective impact approaches, planners are also skilled at working with diverse policymaking stakeholders to manage discourses and garner support for planning of land use activities.

**G.** **Civil, Construction, and Environmental Engineering**

Some of the solutions to homelessness involve designing and building safe and affordable housing infrastructure. However, people experiencing homelessness also experience deprivation of basic services that can be considered human rights, such as clean water, and access to sanitation and hygiene resources. Both the construction of housing infrastructure and the design of systems for water delivery, water treatment, sanitation, and solid waste management fall within the civil, construction, and environmental engineering fields.

Contributions from construction engineering and management focus on two potential areas to tackle housing affordability and address homelessness [76,77]—the mapping of value streams (i.e., visualizing supply chains converging to a project, and the processes used to deliver a product or service), and the use of innovative design methods to deliver value while keeping costs in check. In addition, civil and environmental engineers work closely with city and county officials on permitting requirements for water, sanitation, and solid waste systems and engage with community members at town halls and other meetings on development or redevelopment projects.

Many people experiencing homelessness live alongside water bodies [78,79,80] and in canyons and other softscape environments, where they lack access to water and sanitation systems and where their use of surface water and the practice of open defecation can increase health risks to themselves and others [40,81,82]. The implementation of sanitation solutions in softscape environments is a major challenge due to the more remote locations of many encampments and the reality that access to sanitation is needed 24 h a day, not just during daylight hours. In addition, people experiencing homelessness participate in solid waste management systems and the informal recycling market [83], which is another example of a system designed by environmental engineers. These systems must be re-designed through interdisciplinary approaches (e.g., using community-based participatory methods) to be more inclusive of the needs of people experiencing homelessness, especially in environments where clean water, sanitation, and solid waste collection are lacking.

**H.** **Environmental Design**

Homeless shelters are designed to prioritize emergency housing. This is coupled with public, moralistic views that comfortable shelter environments could potentially dis-incentivize people experiencing homelessness from taking actions to break the cycle of homelessness (e.g., seeking permanent housing, employment) [84]. Consequently, current shelter environments have more in common with the penal sector, where the intention is to reinforce a “no return” mindset in its temporary occupants. The same strategy extends to the planning of new shelters, which are generally located in marginal areas of the city outside of established communities: an “out of sight, out of mind” policy [85,86].

Environmental design can address these failings through interdisciplinary collaboration. For example, collaborative efforts can examine the implications of administrative and structural design for the chronic cycling of homelessness. Shelters typically adopt a top-down organizational model, framed by rules that dictate who is accepted and who is rejected. Shelters and transitional facilities are organized and designed to serve and cycle through as many people as possible. The users’ views and long-term needs are immaterial (e.g., people experiencing homelessness have little voice in administrative decisions; there is inadequate storage space), because people experiencing homelessness are not supposed to linger in shelters and form roots in places of institutional refuge [87]. Environmental design can enhance evaluation of the efficacy of new shelter models that have emerged over the last 20 years, including spontaneous communities and self-managed encampments [88,89,90,91]. Such efforts build upon the existing research findings of social scientists and behavioral psychologists who have investigated the conditions of the long-term unhoused (e.g., [92]).

Interdisciplinary communication and a common research agenda will enable architects and environmental designers to develop an optimal, community-based template for temporary living that empowers rather than objectifies its occupants. Finally, through interdisciplinary collaboration, environmental design can help to forge design-led research, developed in concert with economists, social scientists, and city planners, to focus on a neighborhood-based shelter strategy that sites living spaces in appropriate residential locations. Preliminary studies suggest that contemporary shelters should be adaptable to changing user demographics, shifting regional demand, and the expectations of the existing resident community.

**I.** **Music and Fine Arts**

In the last two decades, arts practitioners have used the power of arts engagement and participation as a counter to the dehumanizing experience of homelessness. The goal of these efforts is to use artistic expression to create a sense of community and connection. A primary manifestation of these efforts is community-based arts organizations working directly with or engaging people experiencing homelessness.

Arts organizations dedicated to populations that are homeless and vulnerable function along all stages of the homelessness continuum. In the pre-homelessness phase, one preventive measure is early intervention through arts engagement to assist vulnerable populations. An example of this is the David’s Harp Foundation, an organization in San Diego focused specifically on youth experiencing homelessness, offering opportunities in music education, sound engineering, and multimedia production [93]. San Diego’s Voices of Our City Choir, a singing ensemble serving people experiencing homelessness, functions in both the homelessness and post-homelessness phases of the continuum by engaging the members of the choir through music and providing help in connecting them with resources. In the post-homelessness phase, the support system of the choir membership has a positive impact on mitigating the chronic cycle of homelessness [94].

Although research in arts intervention for people experiencing homelessness is sparse, it is growing. In their interpretive meta-synthesis, Murphy and Alexander (2020) found that major themes in arts-based intervention programs were healing, advocacy, and self-empowerment [95]. Arts are particularly helpful in bridging the connection between practitioners and researchers, as demonstrated by organizations such as With One Voice [96] and the National Alliance for Music in Vulnerable Communities [97], who focus on sharing knowledge, experience, and research. The growth of this research and development of these organizations illustrates the interest in arts engagement and participation as an integral piece in the puzzle that is addressing the issue of homelessness.

**J.** **Mass Media and Communication**

Mass media plays an important role in communicating about homelessness, including social narratives that can be potentially damaging to people experiencing homelessness and the communities that serve them [98,99,100]. Mass media and communications scholars can contribute to solutions-based approaches to homelessness in several ways, including correcting false narratives about who is experiencing homelessness [67]. Becoming more conscious of the impact of textual and visual messaging has the power to more fully engage community and policymaking stakeholders with the diverse lived experiences of people experiencing homelessness. In doing so, policymaking stakeholders may be more likely to empathetically engage people experiencing homelessness as partners in solution building.

Media scholars can focus on the unique set of circumstances embodied by each individual and shared through stories to identify case-based solutions. Centering research on insider perspectives (the storytellers) values and respects their positionality, elevates the narratives that the storytellers generate into community and policy dialogue, and recognizes those voices as the authority on their situation. Applying this more conscious, insider-centered approach in telling the stories of homelessness creates a framework for this collective approach that has the potential to both transform perceptions and re-imagining solutions. Media and communications scholars contribute to collective efforts to address homelessness by amplifying authentic narratives in a way that can initiate dialogue to better understand, prevent, and reduce homelessness.

## 3. Conclusions

The stubbornly high number of individuals and families experiencing homelessness and the identification of homelessness as the top priority in Southern California accentuate the need for an integrated interdisciplinary approach to address this complex issue. Using a collective impact model, our interdisciplinary approach centralizes the efforts of researchers and practitioners under a shared community agenda.

Our approach requires building institutional capacity (structural and cultural), building connections, participation, and trust with a wide variety of academic and community collaborators, and a long-term dedication to our goals. Building a backbone institution requires a dedicated staff and director, a data repository, and the monitoring and evaluation of research projects and their recommendations. Building institutional capacity has been partially achieved through the creation of SEVI at SDSU which has a dedicated director and has staff support from the Center for Regional Sustainability. SEVI also currently serves as a repository for data from supported projects, and is in the process of monitoring and evaluating research projects focused on homelessness. SEVI has a flat organizational structure with limited levels of hierarchy in order to ensure high levels of interaction and collaboration among different disciplines and with the community. In addition, SEVI has been working on building trust with the broader community by sharing information and findings through its reports, and by engaging with other universities and non-profit organizations to study and collaborate on the issue of homelessness.

SEVI’s objective is to create a partnership where the contributions of all members are valued, heard, and integrated. In the process, trust and collaboration will increase and become part of the norms and culture of the organization. SEVI collaborators believe the culture our partnership will foster will encourage innovation and experimentation.

### Future Goals

As our model grows, we build on our current successes and scale up our three original objectives: (1) making evidence-based decisions, (2) building a community-based participatory approach, and (3) training a workforce skilled in interdisciplinary approach.

In order to empower policy makers, public institutions, service providers, and people experiencing homelessness, our model supports collaboration on projects that are interdisciplinary in nature and are based on qualitative and/or quantitative data collection methods. In the future, our backbone institution (SEVI) will seek funding to support interdisciplinary research that collects original data and that builds on reliable preexisting datasets.

In addition, SEVI will build a community based participatory approach by investing more in building trusted relationships with community entities such as public institutions, service providers, and people experiencing homelessness. Participatory meetings will take place among representatives from various disciplines to determine our current and future agenda, and evaluate success and progress in reaching shared metrics.

Finally, researchers at SDSU have been teaching and engaging their students in interdisciplinary projects around homelessness, thus training future members of the workforce. With more funding and time, new cross-disciplinary courses will be created to engage and train students. In addition, in collaboration with public agencies, interdisciplinary programs will be developed to train agency workforces and open up opportunities through internships for students to apply what they learn to improve health and reduce the chronic cycle of homelessness.

## Data Availability

Not applicable.
